# Supplementary material for: Identification of photoperiod-induced specific miRNAs in the adrenal glands of Sunite sheep (Ovis aries)
Source: Front Vet Sci. 2022 Jul 22;9:888207. doi: 10.3389/fvets.2022.888207 (PMC9354845; doi:10.3389/fvets.2022.888207)
Supplement: Supplementary file 1 [file Data_Sheet_1.ZIP › Supplementary file/miRDeep2 file.zip!/miRDeep2 file/Novel_Structure/SPLP42A3/Novel_386.pdf]

| | | |
|---|---|---|
| Provisional ID | : | NC_019464.2_15761 |
| Score total | : | 200 |
| Score for star read(s) | : | 3.9 |
| Score for read counts | : | 192.8 |
| Score for mfe | : | 1.7 |
| Score for randfold | : | 1.6 |
| Score for cons. seed | : | |
| Total read count | : | 390 |
| Mature read count | : | 389 |
| Loop read count | : | 0 |
| Star read count | : | 1 |

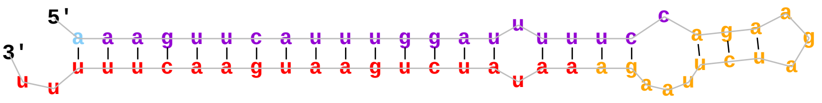


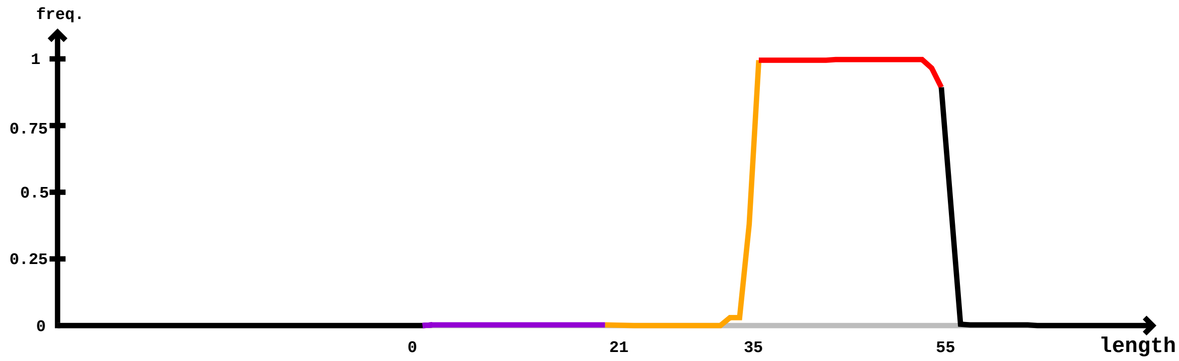


```
                         Star                            Mature
5'- uugucauaaaaggguaaauacuguugaguuggucaaaaaguucauuuggauuuuuccagaagaucuuaagaaauaucugaaugaacuuuuuggccaacccgauuuauaauuc  -3'  obs
    uugucauaaaaggguaaauacuguugaguuggucaaaaaguucauuuggauuuuuccagaagaucuuaagaaauaucugaaugaacuuuuuggccaacccgauuuauaauuc       exp
    ......................((((.((((((((((((((((((((((((.((((.(((...)))...)))).)))))))))))))))))))))))).)))).........   reads   mm   sample
    .....................................aaguucauuuggGuuuuucc.......................................................   1       1    seq
    ....................................................................agaaaAaucugaaugaacuuu.......................   1       1    seq
    ....................................................................agaaaAaucugaaugaacuuuu......................   3       1    seq
    ....................................................................agaaaAaucugaaugaacuuuuu.....................   8       1    seq
    ......................................................................aaaAaucugaaugaacuuu.......................   12      1    seq
    ......................................................................aaaAaucugaaugaacuuuu......................   26      1    seq
    ......................................................................aaaAaucugaaugaacuuuuu.....................   78      1    seq
    ......................................................................aaaAaucugaaugaacuuuuug....................   24      1    seq
    ......................................................................aaauaucugaaugaacuuuuug....................   1       0    seq
    .......................................................................aaAaucugaaugaacuuuuu.....................   247     1    seq
    .......................................................................aauaucugaaugaacuuuuugg...................   1       0    seq
    ...............................................................................aaugaacuuuuuggccaaccc............   1       0    seq
```
